# Estrogen deficiency in ovariectomized rats: can resistance training re-establish angiogenesis in visceral adipose tissue?

**DOI:** 10.6061/clinics/2016(09)08

**Published:** 2016-09

**Authors:** Camila do Valle Gomes-Gatto, Fernanda Oliveira Duarte, Uliana Sbeguen Stotzer, Maria Fernanda Cury Rodrigues, Sérgio Eduardo de Andrade Perez, Heloisa Sobreiro Selistre-de-Araujo

**Affiliations:** Universidade Federal de São Carlos; I Laboratório de Bioquímica e Biologia Molecular; II Departamento de Ciências Fisiológicas, Laboratório de Fisiologia do Exercício; III Departamento de Educação Física e Motricidade Humana, Laboratório de Nutrição e Metabolismo Aplicados ao Exercício, São Carlos/SP, Brazil

**Keywords:** Capillary Density, Ovariectomy, Resistance Training, VEGF-A

## Abstract

**OBJECTIVE:**

The purpose of this study was to investigate the effects of resistance training on angiogenesis markers of visceral adipose tissue in ovariectomized rats.

**METHOD:**

Adult Sprague-Dawley female rats were divided into four groups (n=6 per group): sham-sedentary, ovariectomized sedentary, sham-resistance training and ovariectomized resistance training. The rats were allowed to climb a 1.1-m vertical ladder with weights attached to their tails and the weights were progressively increased. Sessions were performed three times per week for 10 weeks. Visceral adipose tissue angiogenesis and morphology were analyzed by histology. VEGF-A mRNA and protein levels were analyzed by real-time PCR and ELISA, respectively.

**RESULTS:**

Ovariectomy resulted in higher body mass (*p*=0.0003), adipocyte hypertrophy (*p*=0.0003), decreased VEGF-A mRNA (*p*=0.0004) and protein levels (*p*=0.0009), and decreased micro-vascular density (*p*=0.0181) in the visceral adipose tissue of the rats. Resistance training for 10 weeks was not able to attenuate the reduced angiogenesis in the visceral adipose tissue of the ovariectomized rats.

**CONCLUSION:**

Our findings indicate that the resistance training program used in this study could not ameliorate low angiogenesis in the visceral adipose tissue of ovariectomized rats.

## INTRODUCTION

The decline in estrogen levels that occurs in postmenopausal women is closely related to obesity, mainly due to the redistribution of subcutaneous fat to the visceral compartment (intra-abdominal) 1. In turn, obesity, in particular visceral adiposity, is associated with chronic inflammation in adipose tissue (AT), which can cause obesity-associated comorbidities such as insulin resistance and cardiovascular diseases in rodents and humans 2,3.

Studies have demonstrated that postmenopausal women have enlarged adipocytes and that the lipolytic activity in these adipocytes is high, which may explain why postmenopausal women have higher systemic levels of free fatty acids 4. Furthermore, Stubbins et al. 5 showed that ovariectomized (Ovx) female mice have hypertrophied adipocytes, creating hypoxia that can worsen the inflammatory environment. Therefore, it is evident that one of the ways that estrogen modulates adiposity is by altering adipocyte size.

AT is highly vascularized and each adipocyte is surrounded by one or more capillaries, ensuring a close connection is maintained between adipocytes and vessels 6,7. The maintenance of homeostasis in this tissue is of great importance due to its role in supplying nutrients and oxygen to adipocytes 6,7. Blood vessels also provide adipose precursor and stem cells that control the mass and function of AT 6. The expansion and remodeling of AT occurs through hyperplasia or hypertrophy of fat cells and therefore requires angiogenesis 6,7.

Angiogenesis requires the expression of several angiogenic factors 8. Among them, vascular endothelial growth factor A (VEGF-A), which is highly expressed in adipose tissue, is postulated as the major angiogenic factor associated with AT growth, remodeling and metabolic functions. The role of VEGF-A in angiogenesis is mainly modulated through vascular endothelial growth factor receptor-2 (*VEGF-R2*) signaling 6-8. This signaling results in increased vascular density in white adipose tissue (WAT) 8,9.

There is evidence that adipose tissue vascularization is deficient and VEGF-A levels are reduced in obesity 6,10 and that these factors may trigger insulin resistance 8. A study conducted by Elias et al. 8 showed that VEGF-A overexpression can reduce adipocyte hypertrophy and increase capillary density. Despite the importance of estrogen in AT function, little is known about the influence of ovarian hormone deficiency, especially in vasculature 11,12. However, a study conducted by Xu et al. 11 demonstrated that ovariectomy increases periaortic and intra-abdominal fat accumulation and VEGF-A and hypoxia-inducible factor 1-alpha (HIF-1a) expression levels.

Although estrogen replacement (ER) has been associated with some beneficial effects, such as reductions in body mass and adipocyte inflammation, the use of ER is not universally accepted, mainly due to its contraindication in some patients, low patient compliance, patients’ fear of and aversion to side effects, and the associated long-term risks for some types of cancer 13. Therefore, physical exercise has been highlighted as an efficient strategy for the prevention and treatment of the deleterious effects associated with menopause, such as metabolic dysfunction, loss of bone mineral density, diabetes, impairment of muscle function, increased inflammation and obesity 14,15.

The effect of aerobic exercise training on AT angiogenesis has been investigated and studies have reported that exercise can change VEGF-A gene expression patterns in AT 16. In fact, a 9-week treadmill exercise training period increased the density of endothelial cells in visceral (epididymal) AT and up-regulated VEGF-A and *VEGF-R2* mRNA expression in AT stromal vascular cells of young male rats 15,16. Disanzo et al. 15 observed that an 8-week treadmill exercise training period increased VEGF-A gene expression in intra-abdominal AT in rats and decreased lactate levels, an indicator of hypoxia.

Thus, aerobic exercise training may increase angiogenesis, alleviate vasoconstriction and increase blood flow in AT, reducing hypoxia and chronic inflammation associated with obesity 14. In recent years, resistance training (Rt) has been suggested as an important tool to prevent the deleterious physiological and metabolic changes promoted by menopause,including increases in abdominal fat and decreases in lipid metabolism 17,18. However, compared to aerobic exercise, studies evaluating the effects of Rt on angiogenesis in adipose tissue of Ovx rats are scarce.

Therefore, we investigated the effects of ovariectomy and Rt on angiogenesis markers in adipose tissue. Based on the results described above, we hypothesized that Rt attenuates the down-regulation of angiogenesis caused by ovariectomy in rat visceral AT.

## METHODS

### Animals, allocation and ethics

Twenty-four 7-week-old Sprague–Dawley rats (220±12 g) were obtained from the breeding colony of the State University of São Paulo (UNESP, Araraquara, SP, Brazil). The rats were housed in polypropylene cages (three rats/cage) at a controlled temperature of 22±2°C under a 12-h light/12-h dark cycle with food (standard rodent chow) and water provided ad libitum. This research was approved by the Committee of Experimental Animals of the Federal University of São Carlos (protocol no. 008/2010), and all animal procedures were conducted in accordance with the Guide for Care and Use of Laboratory Animals 19.

### Experimental groups

**Figure 1:**
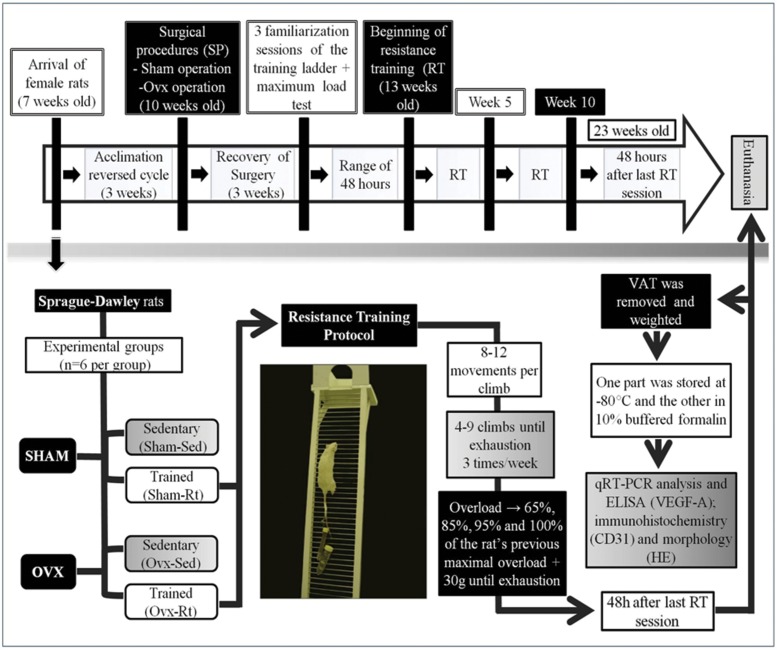
Summary of experimental design. Representative figure of the experimental study design from the arrival of the animals at the laboratory vivarium until the day of euthanasia. VAT=visceral adipose tissue.

A schematic representation of the experimental design is presented in Figure 1. The rats were randomly distributed into 4 experimental groups (n=6/group): (i) sham-sedentary (Sham-Sed), (ii) ovariectomized sedentary (Ovx-Sed), (iii) sham-resistance training (Sham-Rt), and (iv) ovariectomized resistance training (Ovx-Rt). The sedentary animals (Sham-Sed and Ovx-Sed) were kept in their cages over the whole experimental period without any type of exercise. The Ovx animals (Ovx-Sed and Ovx-Rt) had their ovaries removed. The trained animals (Sham-Rt and Ovx-Rt) underwent a 10-week resistance training program, which was initiated at the same time for each group and is described below.

### Ovariectomy and sham surgery

Ovariectomy and sham surgery were performed when the rats reached 10 weeks of age (body mass of 250 g) according to the technique described by Kalu 20. A mixture of ketamine and xylazine (61.5-7.6 mg/kg, intraperitoneal injection) was used as an anesthetic. The sham-operated rats underwent the surgical procedure, but the ovaries were not removed. The ovaries were removed only from the Ovx animals. All animals that underwent surgical procedures had 3 weeks of recovery before starting Rt. All animals were euthanized 92 days after the surgical procedure.

### Resistance exercise training protocol

The Rt protocol was adapted from that of Hornberger and Farrar 21 according to the needs of the current investigation. During the 10 weeks of Rt, climbing sessions were performed 3 times per week (Figure 1). Initially, the rats were adapted to the Rt protocol, which required them to climb a vertical ladder (1.1×0.18 m, 2-cm grid, 80° incline) with weights attached to their tails. The size of the ladder required the animals to perform 8-12 movements per climb. The load apparatus was attached to the tail by wrapping the proximal portion of the tail with a self-adhesive foam strip. A Velcro strap was wrapped around the foam strip and fastened. With the load apparatus attached to the tail, each rat was placed at the bottom of the ladder and familiarized with climbing. If necessary, a stimulus was applied to the animal’s tail with tweezers to initiate the movement. At the top of the ladder, the rats reached a housing chamber (20×20×20 cm), where they were allowed to rest for 120 s. This procedure was repeated until the rats voluntarily climbed the ladder three consecutive times without stimuli.

The first training session started three days after this familiarization and consisted of four to eight ladder climbs with progressively heavier loads. For the initial climb, each animal carried a load that was 75% of its body mass. Subsequently, weight increments of 30 g were added until the load did not allow the animals to climb the entire length of the ladder. The highest load successfully carried over the entire length of the ladder was considered the rat’s maximal carrying capacity for that training session.

The next training sessions consisted of four ladder climbs with 65, 85, 95, and 100% of the rat’s previous maximal carrying capacity, as determined in the previous session. During subsequent ladder climbs, an additional 30-g load was added until a new maximal carrying capacity was determined.

### Euthanasia and tissue sampling

Animals were euthanized by decapitation 48 h after the last resistance exercise session. Immediately after euthanization, the uterus and visceral adipose tissue (VAT) (mesenteric and omental) were removed as described by Duarte et al. 22. The uterus was weighed to confirm ovariectomy or sham surgery. A portion of the VAT was frozen in liquid nitrogen and stored at -80°C for qPCR analysis and ELISA. Another portion was stored in 10% buffered formalin for histological analysis.

### Isolation of RNA and qRT-PCR

Total RNA was extracted from approximately 100 mg of frozen VAT using TRIZOL reagent (Invitrogen Corporation, California, USA) according to the manufacturer’s specifications. The purity and concentration of the RNA samples were determined using a Nanodrop spectrophotometer. The integrity of the RNA was verified via agarose gel (2%) electrophoresis. Reverse transcription was performed using M-MLV reverse transcriptase and oligo(dT) (Promega Corporation, Madison, WI, USA). SsoFast™ EvaGreen^®^ Supermix EVAGreen (Bio-Rad) was used for quantitative RT-PCR. All samples were analyzed in duplicate. Gene-specific primers are listed in Table 1. The thermal cycling parameters were 95°C for 30 s followed by 40 cycles of 95°C for 15 s and 57–61°C for 30 s. Large ribosomal protein (RPLPO) was used as an endogenous control and the relative expression levels of the quantitative RT-PCR products were determined using the ΔΔCt method 23.

### ELISA

Approximately 100 mg of the VAT was homogenized in lysis buffer containing 10 mM Tris (pH 7.4), 100 mM NaCl, 1 mM EDTA, 1 mM EGTA, 1% Triton X-100, 10% glycerol, 0.1% SDS, 0.5% deoxycholic acid, 0.2 mM PMSF, and protease inhibitors (Sigma, P8340). Homogenates were centrifuged at 4˚C and 13,000 x g for 20 min, and supernatants were assayed for total protein concentration (BCA Protein Assay Kit; Pierce Biotechnology, Rockford, Illinois, USA). VEGF-A expression was assessed using a commercially available ELISA VEGF-A mini Kit (*Rat VEGF mini ELISA Development kit*, PeproTech) according to the manufacturer’s specifications. The range of detection for the kit was 32-2000 pg/mL. Optical density reading was performed at a wavelength of 405 nm in a plate reader (SpectraMax M5, Molecular Devices). All samples and standards were measured in duplicate and the VEGF-A levels in the samples were normalized to the total protein concentration and expressed in ng of VEGF-A per mg of protein.

### Immunohistochemical and morphometric analysis

VAT samples were fixed for 24 h in formalin, embedded in paraffin, and sectioned. For immunohistochemical detection (IHC), the sections were deparaffinized and incubated overnight (4˚C) with a monoclonal mouse anti-CD31 antibody (Abcam: ab24590, diluted 1:100). A peroxidase-labeled secondary antibody (ImmPRESS kit, Vector Laboratories, Burlingame, CA) was used for signal amplification and was revealed using DAB chromogen substrate. The sections were counterstained in Mayer’s hematoxylin. A morphometric study of adipocyte size was performed in five-micron-thick VAT sections stained with hematoxylin-eosin (HE). Slides were randomly digitized under a light microscope for HE (10 fields per animal) and for IHC (6-10 fields per animal). *Image-Pro^®^ Plus 6.0* was used (Media Cybernetics, Silver Spring, MD, USA) for quantification of adipocyte area (μm^2^) and quantification of microvascular density through DAB-positive pixels for CD31.

### Statistical analysis

To check the normality and homoscedasticity of the data, the Shapiro-Wilk test and Levene’s tests were used, respectively. A two-way analysis of variance (ANOVA) was used to compare the variables Rt and ovariectomy. Tukey’s post hoc test was used in the event of a significant (*p*<0.05) ratio. All results are presented as the mean±standard error mean (SEM). STATISTICA 7.0 (Stat Soft) was used for all statistical comparisons.

## RESULTS

**Figure 2:**
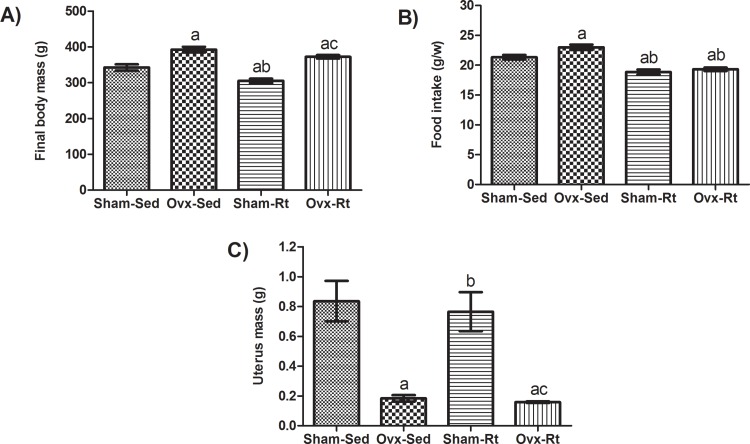
Body mass, food intake and uterus mass. **(A)** Average body mass, **(B)** average food intake, and average uterus mass **(C)** after the experimental period. n=6 rats per group. Data are shown as the mean±SEM (*p*<0.05). **a.** Different from Sham-Sed; **b.** Different from Ovx-Sed; **c.** Different from Sham-Rt. g=gram, w=week.

After the 10-week experimental period, the Ovx animals all showed increased body mass (*p*=0.0003), regardless of whether they engaged in Rt (Figure 2A). Food intake was increased (*p*=0.0400) following Ovx, but it was decreased following Rt (*p*=0.0001) (Figure 2B).

Differences in vaginal smear test results (*data not shown*) and decreases in uterus mass in the Ovx-Sed rats compared to the Sham-Sed rats indicated that the ovariectomies were successful (Figure 2C).

**Figure 3:**
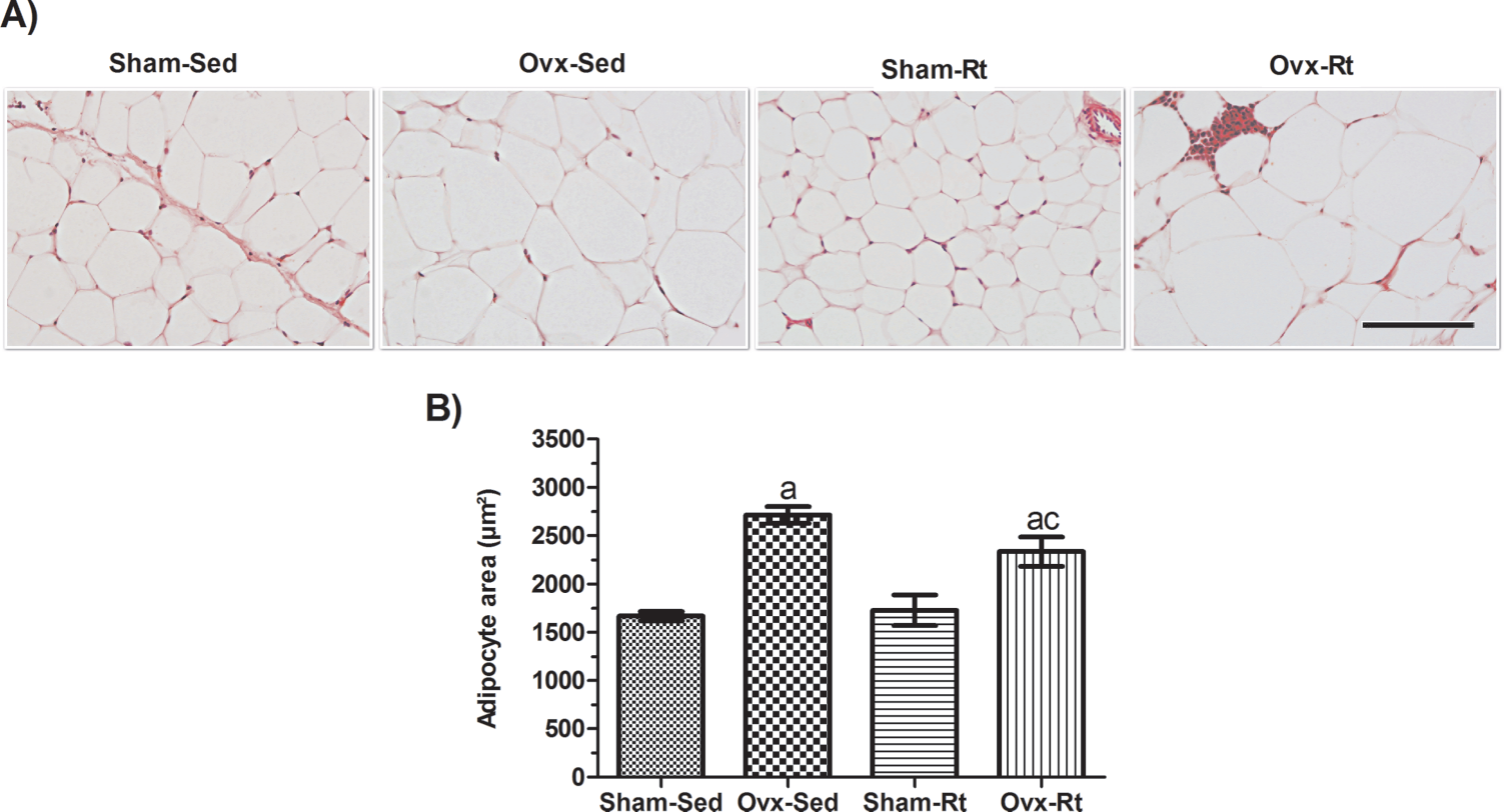
Effects of ovariectomy and resistance training on adipocyte size. **(A)** Representative hematoxylin and eosin (HE)-stained sections (5-μm-thick) of visceral AT fat pads from female rats (20 μm; original magnification x400). **(B)** HE-stained sections were analyzed with an image analysis system, and adipocyte size was quantified. Sham sedentary (Sham-Sed), ovariectomized sedentary (Ovx-Sed), sham resistance training (Sham-Rt), and ovariectomized resistance training (Ovx-Rt) groups are shown. Data are shown as the mean±standard error of the mean (SEM). n≤6 per group, *p*<0.05. **a.** Different from Sham-Sed; **b.** Different from Ovx-Sed; **c.** Different from Sham-Rt.

Adipocyte size was increased in the Ovx rats (Figure 3A and B, *p*=0.0003) compared to the Sham-Sed rats, and Rt did not significantly prevent this increase. Thus, no difference was observed in adipocyte size between the Sham-Sed and Sham-Rt groups.

**Figure 4:**
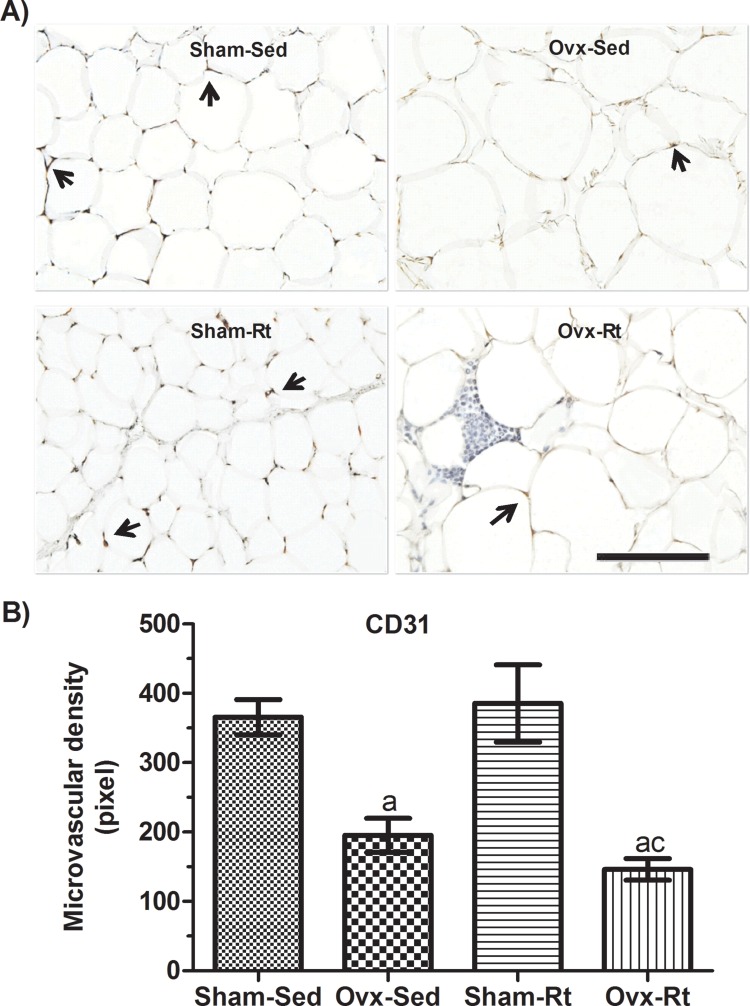
Effects of ovariectomy and resistance training on microvascular density. **(A)** Representative photomicrographs with endothelial cells visualized by immunohistochemistry with an anti-CD31 antibody (20 μm; original magnification x400) and **(B)** quantification of endothelial cell density in visceral adipose tissue fat pads. Sham sedentary (Sham-Sed), ovariectomized sedentary (Ovx-Sed), sham resistance training (Sham-Rt), and ovariectomized resistance training (Ovx-Rt) groups are shown. Black arrows indicate capillary vessels. Data are shown as the mean±standard error of the mean (SEM). n≤6 rats per group, *p*<0.05. **a.** Different from Sham-Sed; **b.** Different from Ovx-Sed; **c.** Different from Sham-Rt.

Microvascular density in VAT was reduced in the Ovx rats (*p*=0.0181) compared to the Sham-Sed rats (Figure 4A and B); however, there was no difference between the Sham-Sed and Sham-Rt groups or between the Ovx-Sed and Ovx-Rt groups. In addition, capillary density in the Ovx-Rt group was lower than that in the Sham-Rt and Sham-Sed groups (*p*=0.0016 and *p*=0.0032, respectively).

**Figure 5:**
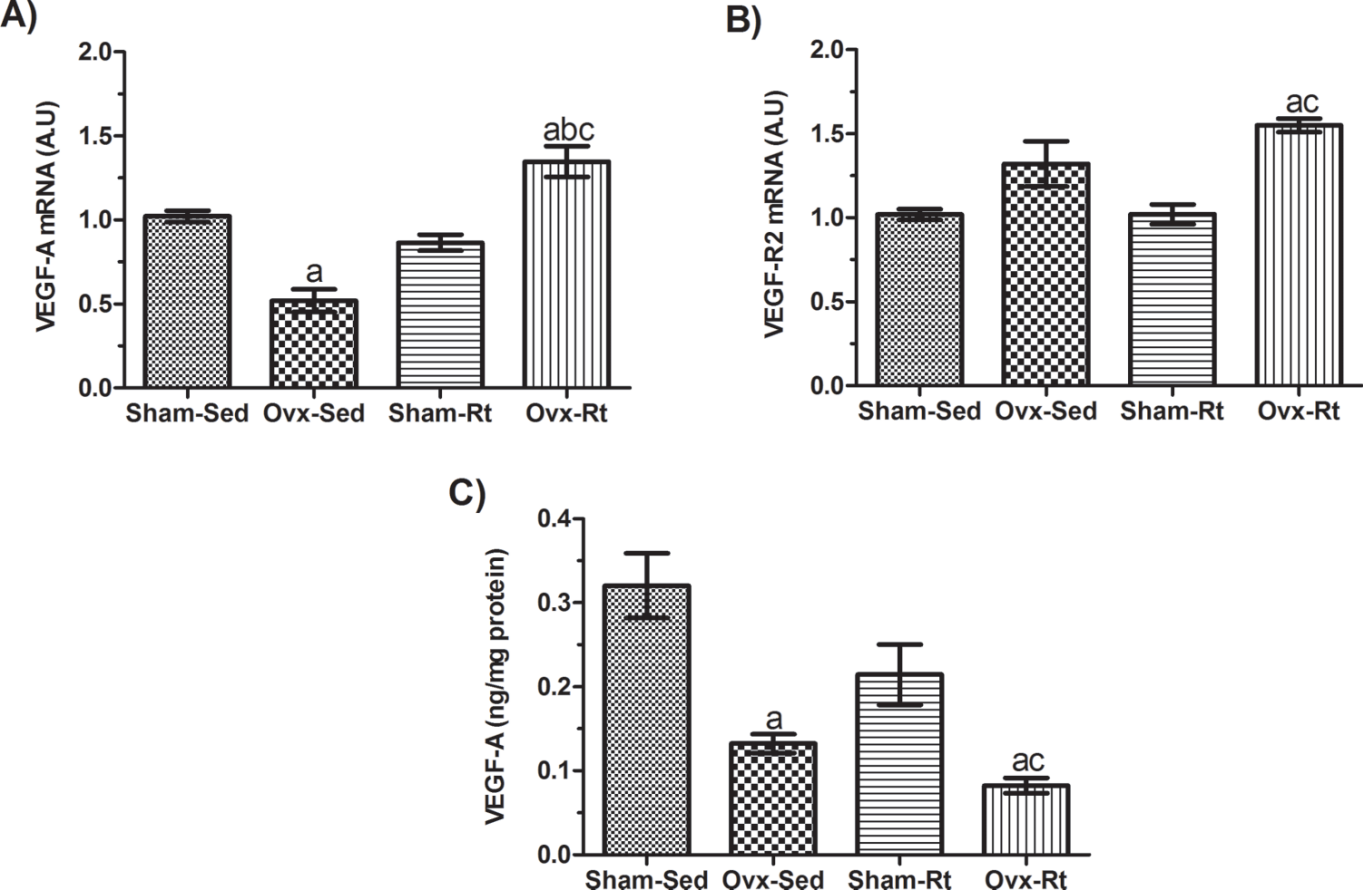
Expression of VEGF-A **(A)** and VEGF-R2 **(B)** mRNA levels and VEGF-A protein levels **(C)** in the visceral adipose tissues of female rats following ovariectomy and Rt. RPLPO (large ribosomal protein) was used as an endogenous gene control. VEGF-A protein expression was normalized to the total protein concentration and was therefore expressed in ng of VEGF-A per mg of protein. Sham sedentary (Sham-Sed), ovariectomized sedentary (Ovx-Sed), sham resistance training (Sham-Rt), and ovariectomized resistance training (Ovx-Rt) groups are shown. Data are shown as the mean±standard error of the mean (SEM). n≤6 rats per group, *p*<0.05. **a.** Different from Sham-Sed; **b.** Different from Ovx-Sed; **c.** Different from Sham-Rt.

Ovariectomy reduced VEGF-A mRNA (Figure 5A, *p*=0.0004) and protein levels (Figure 5C, *p*=0.0009), but not VEGF-R2 gene expression (Figure 5B), compared to controls. There was no difference between the Sham-Sed and Sham-Rt groups. Rt up-regulated VEGF-A mRNA expression only in the Ovx animals (Figure 5A, *p*=0.0002), with no changes observed in VEGF-R2 mRNA (Figure 5B) and protein levels (Figure 5C).

## DISCUSSION

Postmenopausal status with estrogen insufficiency is associated with increased visceral adiposity, inflammation and metabolic abnormalities 1,2,24. Ovx animals have been used as models for studying the effects of ovarian hormone deficiency 18,25. Evidence from rodent models has demonstrated that ovariectomy promotes obesity and its metabolic complications, in particular insulin resistance 26,27.

It has been recognized that angiogenesis has protective effects on AT metabolism by reducing adipocyte size, hypoxia and inflammation in all stages of AT expansion during obesity 8. VEGF-A overexpression during angiogenesis is able to reduce adipocyte hypertrophy and body mass in obesity 8. In this context, regular exercise has been shown to regulate pro-angiogenic pathways through the induction of HIF-1a, which induces the transcription of VEGF-A 28,29.

In the present study, ovariectomy decreased angiogenesis in the VAT of rats. Additionally, Rt failed to attenuate the decreased angiogenesis caused by the surgery.

Sex steroids such as estrogen are known to regulate the development and function of AT in a protective manner 2. Thus, postmenopausal women show an increase in AT mass, especially for visceral fat, contributing to deficient vascularization in this tissue 4. Additionally, estrogen plays an important role in body composition by suppressing WAT accumulation through reductions in fatty acids, triglyceride synthesis and lipogenesis 4,18. In our study, ovariectomy resulted in increases in both body mass and adipocyte hypertrophy in VAT. Stubbins et al. 5 showed an increase in adipocyte lipolysis in Ovx rats 10 wk after surgery, and this increase was associated with larger adipocytes. An expansion in adipocyte size can potentially create a hypoxic environment due to reduced blood flow triggering an inflammatory process in the vascular stromal fraction (VSF) and can even lead to tissue fibrosis 6,30. In an attempt to reverse this scenario, angiogenesis is strongly stimulated by hypoxia and inflammation through the recruitment of important angiogenic factors such as VEGF-A 8, which protect against hypoxia and inflammation in AT 31.

In the current study, VEGF-A mRNA and protein levels were decreased following ovariectomy with no changes in VEGF-R2 mRNA expression. Sung et al. 9 showed that increased blood vessel density obtained from the induction of VEGF-A expression in fat improves adipose function by increasing blood perfusion, decreasing adipocyte size, reducing hypoxia and preventing adipocyte apoptosis in animals fed a high-fat diet. Additionally, Elias et al. 8 indicated that VEGF-A overexpression increased the number and size of blood vessels in AT, providing protection against hypoxia induced by high-fat diet and obesity. At the same time, Luo et al. 32 showed that ER promotes increased proliferation, VEGF-A production and adipogenic differentiation in human adipose tissue-derived stem cells (ADSCs), and also reduces apoptosis *in vitro*. However, Xu et al. 11 found higher VEGF-A levels in the intra-abdominal fat of Ovx rats, which were reversed by ER.

Evidence has shown that when angiogenesis becomes insufficient or does not occur properly, as in the case of a rapid expansion of fat pad through enlargement of existing fat cells, the formation of new capillaries can be compromised 8,30. Zachwieja et al. 29 found that adipose-specific VEGF-A-deficient mice exhibit up to a 40% decline in adipose tissue capillarity. In support of these findings, our results showed that capillary density is reduced by ovariectomy, which is related to a reduction in AT blood flow, with failure in the angiogenic process 33.

It has been shown that the use of ER prevents obesity-related diseases in women, and alternative therapies such as exercise and diet have recently been considered. Exercise training is considered an important tool for regulating body mass and low-grade systemic inflammation in humans and animals 14,15, thus controlling obesity.

In this context, studies have been performed using different physical training protocols as a non-pharmacological intervention to reduce visceral adiposity and to increase VEGF-A gene and protein expression in AT 29,34. It was verified that aerobic training improves adipocyte biogenesis, up-regulates VEGF-A protein and mRNA levels, attenuates macrophage infiltration and decreases hypoxia in WAT 15,24. Together, these findings suggest that aerobic exercise offers protective modulation of VEGF-A levels in AT, at least in those who are overweight or obese 15.

Recent studies have indicated that Rt is beneficial for skeletal muscle, bones and liver 17,18,35. Previous studies conducted by our group have shown that Rt is able to decrease fat accumulation and deposition in the livers of Ovx rats 36 and to reduce the gene expression of molecules related to lipogenesis 17. Speretta et al. 37 showed that 8 weeks of low-volume/high-intensity Rt controls body mass, minimizing increases in retroperitoneal and visceral adipose and producing significant changes in the lipid profiles of obese rats.

However, limited data are available regarding angiogenesis in AT after this type of training. In skeletal muscle, it is well accepted that Rt promotes increased capillary density and angiogenesis 38. Increases in the capillary network play important roles in improving aerobic capacity, facilitating oxygen transport, enhancing conductance, and increasing muscle contraction, thus increasing maximal oxygen uptake and physical conditioning. Karimian et al. 39 demonstrated that Rt performed by climbing ladders does not change the capillary/fiber ratio in flexor hallucis longus (FHL) or soleus muscles but does increase plasma nitric oxide (NO) concentration, an important factor for regulating and enhancing angiogenesis in diabetic animals.

It was observed in the present study that Rt induced marked reductions in food intake but did not decrease body mass in relation to Ovx sedentary rats. It has been established that Rt significantly induces strength gains and increases lean muscle mass without changing total body mass due to the offsetting between lean muscle mass gain and fat mass loss 40,41. Moreover, an effect of Rt on attenuating adipocyte hypertrophy promoted by ovariectomy was not found in our study. It is well established that estrogen decreases adipocyte lipoprotein lipase activity and increases its activity in skeletal muscle during exercise, resulting in changes in the flow of free fatty acids derived from plasma triglycerides into the muscle, probably favoring fat oxidation 42. In fact, studies have shown that estrogen increases the expression of genes involved in fatty acid oxidation, suggesting that this hormone stimulates the use of fatty acids for energy 42,43. Thus, lipid oxidation can be impaired by estrogen deficiency during training, which would explain the results obtained in our study.

In the present study, it was found that Rt up-regulates VEGF-A mRNA expression in the VAT of Ovx rats, but surprisingly, this up-regulation did not result in increased protein levels, even when changes in VEGF-R2 mRNA were observed. Our findings are concordant with the results of Czarkowska-Paczek et al. 34, who also reported a discrepancy between VEGF-A mRNA expression and protein levels in AT following aerobic exercise. These results indicate the importance of evaluating post-transcriptional molecular mechanisms, such as microRNAs (miRNAs) 44, that could be involved in regulating VEGF-A expression in AT in response to physical exercise. In addition, in the present study, Rt did not up-regulate capillary density in Ovx rats and was associated with a decrease in VEGF-A protein levels.

To the best of our knowledge, the present study is the first to demonstrate the effects of Rt on angiogenesis markers in VAT in Ovx rats. The relevant findings of this study indicate that ovariectomy promoted hypertrophy of visceral fat cells and decreased both VEGF-A levels and capillary density in rats. However, the Rt program used in this study failed to ameliorate the reduced angiogenesis observed in the VAT of Ovx rats. These results are relevant for considerations of whether non-pharmacological treatments can serve an alternative choice to drug treatment and support further studies of what exercise protocols serve as good treatment options. In the future, studies of Ovx rats treated with ER should be performed to confirm whether Rt can improve angiogenesis markers in adipose tissue in estrogen insufficiency.

## AUTHOR CONTRIBUTIONS

Stotzer US, Rodrigues MF and Perez SE were responsible for the conception and design of the experiments. Gomes-Gatto CV, Duarte FO and Selistre de Araujo HS were responsible for data acquisition, analysis and interpretation. Gomes-Gatto CV, Duarte FO, Stotzer US, Rodrigues MF, Perez SE and Selistre de Araujo HS were responsible for drafting and revising the manuscript for important intellectual content.

## Figures and Tables

**Table 1:** Oligonucleotide primers used for quantitative RT-PCR.

Genes	Sense primer (5’-3’)	Antisense primer (5’-3’)	Accession n°.
**VEGF-A**	TGAGACCCTGGTGGACATCTT	CACACAGGACGGCTTGAAGA	NM_001110334.1
**VEGF-R2**	CCACCCCAGAAATGTACCAAAC	AAAACGCGGGTCTCTGGTT	NM_013062
**RPLPO**	AGGGTCCTGGCTTTGTCTGTGG	AGCTGCAGGAGCAGCAGTGG	NM_022402.2

VEGF-A, Vascular endothelial growth factor; VEGF-R2, vascular endothelial growth factor receptor-2; RPLPO, large ribosomal protein.
